# Supplementary material for: Physiological Responses of a Grapefruit Orchard to Irrigation with Desalinated Seawater
Source: Plants (Basel). 2024 Mar 9;13(6):781. doi: 10.3390/plants13060781 (PMC10975856; doi:10.3390/plants13060781)
Supplement: Supplementary file 1 [file plants-13-00781-s001.zip › plants-2866106-supplementary.pdf]

Supplementary material

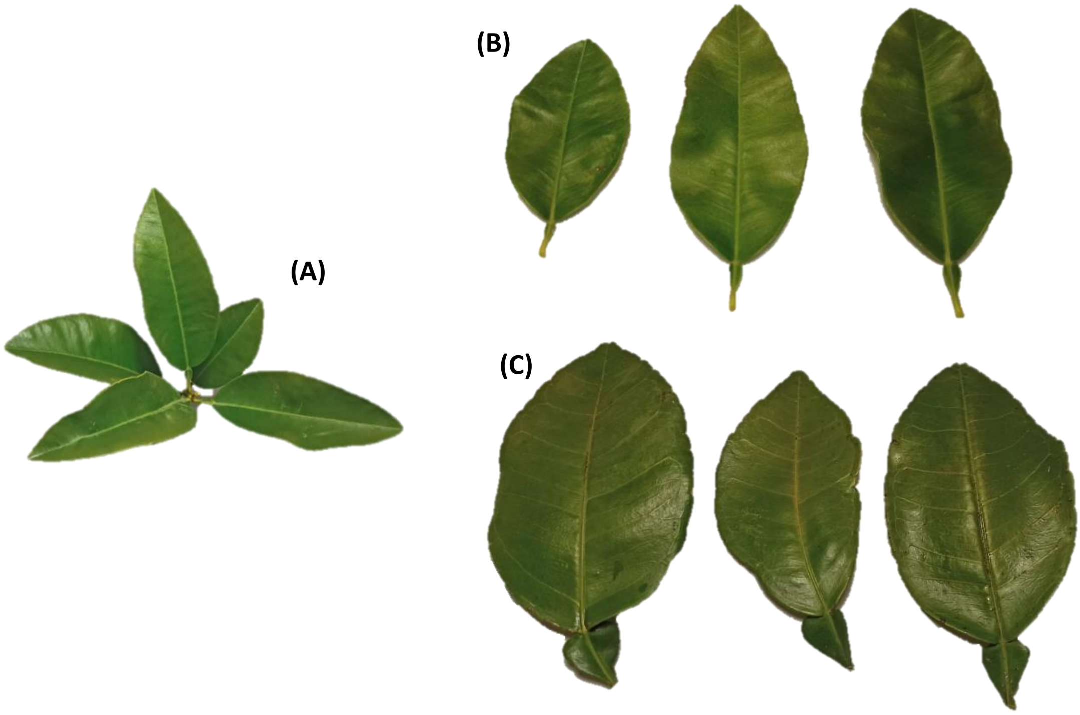

Figure S1. Shape of bud (A), new leaves (B) and old leaves (C) of a grapefruit tree from the orchard.

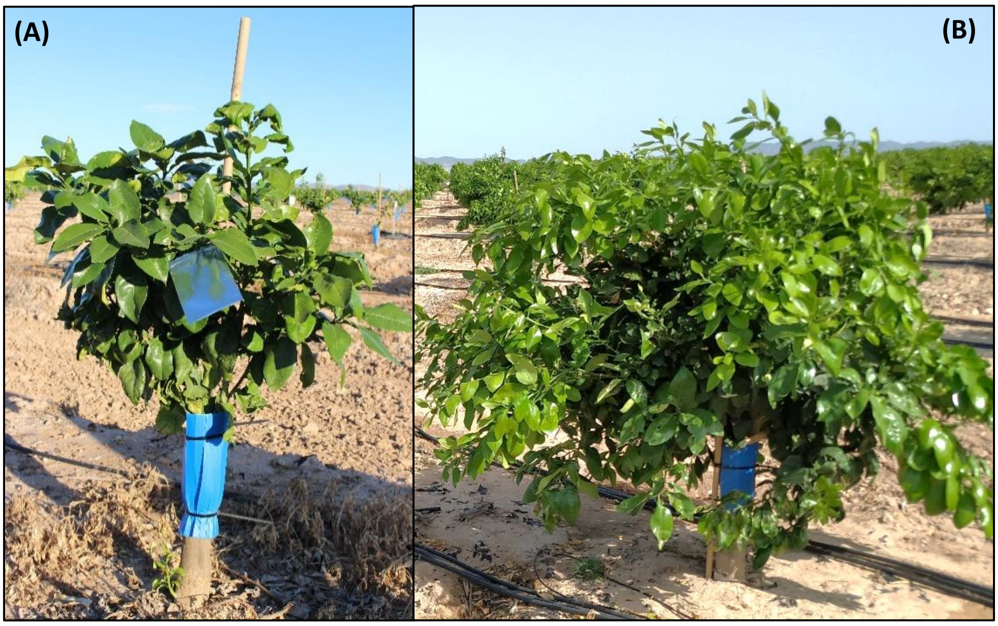

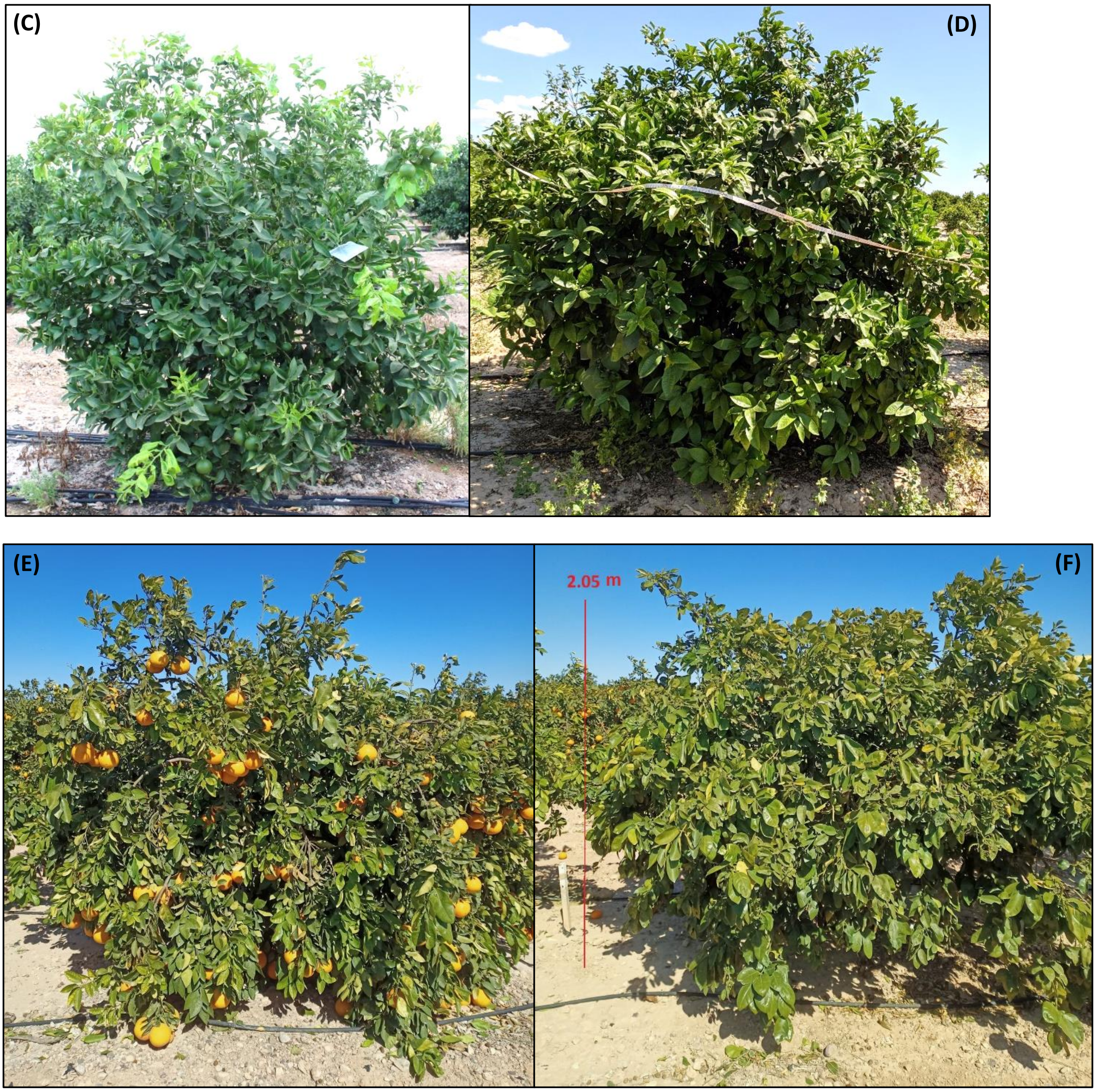

Figure S2. Grapefruit trees in the orchard in November 2019 (A), July 2020 (B), July 2021 (C), May 2022 (D), and December 2022 before (E) and after yield (F), indicating the current average height of the trees.
